# Insight, distress and coping styles in schizophrenia

**DOI:** 10.1016/j.schres.2007.04.030

**Published:** 2007-08

**Authors:** Michael Cooke, Emmanuelle Peters, Dominic Fannon, Anantha P.P. Anilkumar, Ingrid Aasen, Elizabeth Kuipers, Veena Kumari

**Affiliations:** a Department of Psychology, Institute of Psychiatry, King's College London, London, UK; b Section of General Psychiatry, Division of Psychological Medicine, Institute of Psychiatry, King's College London, London, UK

**Keywords:** Insight, Schizophrenia, Coping, Social support, Positive interpretation

## Abstract

**Background:**

The stigma and negative societal views attached to schizophrenia can make the diagnosis distressing. There is evidence that poor insight into symptoms of the disorder and need for treatment may reflect the use of denial as a coping style. However, the relationships between insight and other coping styles have seldom been investigated.

**Method:**

We examined the associations between insight, distress and a number of coping styles in 65 outpatients with schizophrenia (final *n* = 57) in a cross-sectional study.

**Results:**

We found that (i) awareness of symptoms and problems correlated with greater distress, (ii) ‘preference for positive reinterpretation and growth’ coping style correlated with lower distress and with lower symptom awareness (re-labelling), (iii) ‘preference for mental disengagement’ coping style correlated with greater distress and lower awareness of problems, and (iv) ‘social support-seeking’ coping style correlated with greater awareness of illness, but not distress. No relationship occurred between the use of ‘denial’ as a coping style and insight or distress.

**Conclusions:**

Our findings demonstrate that awareness of illness and related problems is associated with greater distress in schizophrenia. However, this investigation has not supported a simple psychological denial explanation for this relationship, as complex relationships emerged between different dimensions of insight and coping styles. The negative association between ‘positive reinterpretation and growth’ and distress suggests that adopting this style may lead to re-labelling symptoms in a less distressing way. Avoidant and isolating styles of coping both appear unhelpful. Psychological interventions should aim to promote more active coping such as discussing a mental health problem with others.

## 1 Introduction

People diagnosed with schizophrenia frequently disagree with their friends, relatives and clinicians about whether they are mentally ill, whether their experiences and behaviours are abnormal, and whether they are in need of psychiatric treatment such as medication. Such disagreements are widely held to reflect poor insight on the part of the patient; insight dimensions typically include awareness of illness, awareness of symptoms, and recognition of the need for treatment, respectively (David, 1990). Poor insight is sometimes seen as just another symptom or manifestation of the disorder (Cuesta and Peralta, 1994). However, another conceptualisation is that poor insight represents an individual response to the diagnosis of schizophrenia.

Schizophrenia is a highly stigmatising disorder (Thornicroft, 2006). Many individuals with this diagnosis feel devalued and discriminated against as a result (Dickerson et al., 2002). Societal, and sometimes medical, views include the belief that it is a chronic, debilitating condition from which individuals have little chance of recovering (Angermeyer et al., 2004). This conceptualisation can be threatening and distressing to those given the diagnosis, and is likely to contribute to the high level of depression experienced by many people with schizophrenia (Mulholland and Cooper, 2000). One way in which individuals might cope with this situation is by denial (for review see Goldbeck, 1997), leading to poor insight.

A link between denial as a coping mechanism and poor insight was suggested shortly after schizophrenia was first delineated, when Mayer-Gross (1920) identified ‘denial of the psychotic experience’ as one of the strategies adopted by patients with schizophrenia, in that, typically, they were unaware of the symptoms of the illness (i.e. lacked insight). The seminal review of Amador et al. (1991) highlighted the denial of illness through psychological coping mechanisms as a potential aetiological model of poor insight in schizophrenia. More recently, poor insight has again been hypothesised to reflect the use of a psychological defence mechanism in the form of denial of illness (Moore et al., 1999). Implicit in all of these models is the concept that denial serves to protect the individual from the distress which acknowledging the presence of illness would cause (Moore et al., 1999).

The psychological denial model predicts that those who deploy denial as a coping strategy will have poorer insight, but will suffer less distress. Indeed, a number of cross-sectional studies support a relationship between higher insight and greater distress, including depression (meta-analysis, Mintz et al., 2003), hopelessness (Carroll et al., 2004), and suicidality (Schwartz and Smith, 2004). Furthermore, longitudinal studies have also shown that as insight increases, both depression (Carroll et al., 1999) and suicidal ideation (Cunningham Owens et al., 2001) worsen. These studies led Schwartz (2001) to hypothesise that there is a chain of causality from insight, to demoralisation, to depression, to suicidality. A study using structural equation modelling (Drake et al., 2004) has also found evidence for the direction of causality proceeding from increasing insight to greater depression.

Although these relationships between insight and distress are consistent with the psychological denial model, they do not test the model explicitly. To test the hypothesis that the use of denial is directly related to poor insight, it is necessary to measure coping styles (including denial) directly. A number of studies have addressed this question, and have found associations between coping styles and insight in schizophrenia. Greater ‘self-deceptive positivity’ (the tendency to give self-reports that are honest but positively biased) has been linked to lower awareness of illness, while greater ‘impression management’ (deliberate positive self-presentation to an audience) has been linked to lower past awareness of illness, its social consequences, and the effects of medication (Moore et al., 1999). These results are interpreted by the authors as suggesting that clinical insight is, at least in part, a function of denial (Moore et al., 1999). However, denial is only one of the many ways that people cope with problems, and the above studies did not investigate other coping styles. More recently, Lysaker et al. (2003a) looked at the relationships between specific dimensions of insight and preference for particular coping styles in schizophrenia. A preference for using ‘escape-avoidance’ as a coping style was related to lower awareness of the consequences of illness, while greater preference for ‘positive reappraisal’ was correlated with lower awareness of symptoms (Lysaker et al., 2003a). However, this study did not look at how these relationships related to distress.

The present study aimed to examine the associations between insight, distress, and coping styles in a sample of people with a diagnosis of schizophrenia. Following the psychological denial model, we hypothesised that poor insight would be associated with both the use of denial as a coping strategy and less distress. We also examined the relationships of insight and distress to 14 other coping styles (see Section 2.2), in addition to the use of denial, as measured by the COPE (Carver et al., 1989). Of these 14 styles, ‘positive reinterpretation and growth’ corresponds most with the ‘positive reappraisal’ dimension assessed by the Ways of Coping Questionnaire (WCQ, Folkman and Lazarus, 1988; used in Lysaker et al., 2003a), while ‘behavioural disengagement’ reflects avoidance. Based on the observations of Lysaker et al. (2003a), we predicted that ‘positive reinterpretation and growth’ and ‘behavioural disengagement’ would both correlate with lower insight, with the latter correlating most strongly with ‘awareness of symptoms’ and the former with ‘awareness of illness’ dimensions within the David's model of insight. Although no study to our knowledge has investigated ‘positive appraisal’ or ‘avoidant’ coping styles in relation to distress in schizophrenia, previous studies in people with physical illnesses suggest reliable associations between ‘approach’ styles of coping and psychological well being (Roesch et al., 2005) and between ‘avoidant’ styles of coping and the severity of depression, anxiety and anger (e.g. Doering et al., 2004). Since patients with schizophrenia have similar illness representations to those with physical health problems (Watson et al., 2006), we predicted that ‘positive reinterpretation and growth’ would correlate negatively, and ‘behavioural disengagement’ positively, with distress in our study sample.

## 2 Materials and methods

### 2.1 Subjects and design

This was a cross-sectional study. Sixty five outpatients with schizophrenia or schizoaffective disorder (diagnosed using the Structured Clinical Interview for DSM-IV Research Version; SCID-P, Spitzer et al., 1994), were included. They were recruited from the South London and Maudsley NHS Trust. All patients were on stable doses of antipsychotic medication for at least three months prior to taking part in this study, and were in a stable (chronic) phase of the illness, living in the local community. Table 1 shows demographic and clinical characteristics of the study sample. The study procedures had the approval of the ethics committee of the Institute of Psychiatry and South London and Maudsley NHS Trust, London. All participants provided written informed consent.

**Table 1:** Demographic and clinical characteristics of patients

Patient characteristic		Mean (SD)	Range
Gender	(Male/Female)	46/19 (71%/29%)	
Age (yrs)		38.92 (10.17)	19–62
Diagnosis (*n*)	Schizophrenia	59	
	Schizoaffective Disorder	6	
Medication (*n*)	Atypical antipsychotic	50	
	Typical antipsychotic	14	
	Mood stabiliser	7	
PANSS symptoms	Positive	16.8 (4.9)	7–28
	Negative	18.3 (5.0)	7–32
	General	32.5 (6.5)	18–48
	Total	67.6 (13.8)	39–108

PANSS: Positive and Negative Syndrome Scale (Kay et al., 1987).

### 2.2 Clinical assessment

#### 2.2.1 Insight

Insight was assessed using two multi-dimensional measures based on David's (1990) model of insight: the Schedule for the Assessment of Insight — Expanded (SAI-E, Kemp and David, 1997) and the Birchwood insight scale (BIS, Birchwood et al., 1994). The SAE-I is a researcher-rated measure and the BIS is a self-report measure.

The SAI-E measures the dimensions of re-labelling of unusual mental events as abnormal, awareness of illness, and recognition of the need for treatment included in the original SAI (David et al., 1992). The SAI-E adds items regarding awareness of psychological/emotional changes, awareness that there is something wrong, awareness of the negative effects of mental illness, and attribution of symptoms to a mental illness. Each item is scored using a set of criteria listed in the text of the response form completed by the interviewer. It is not clear from the published literature if and how these items should be incorporated into the original SAI dimensions, and studies have instead used the total SAI-E score.

Like the SAI-E, the BIS is based on David's (1990) model of insight and measures the same three core dimensions of insight as the original SAI (David et al., 1992). Each item is rated as ‘agree’, ‘disagree’ or ‘unsure’, giving an item score of 1 for unsure, and 0 or 2 for agree and disagree, depending on whether agreement with the statement indicates good insight (the items are counterbalanced for response valence). As all participants of this study were outpatients, item 4 of the BIS “My stay in hospital is necessary” was excluded. The remaining three items from the ‘awareness of need for treatment’ dimension were used to calculate a score for this subscale with equal weight to the other two subscales, allowing a total score to be calculated which has the same range (0–12) as the full scale.

In addition, the PANSS (Positive and Negative Syndrome scale; Kay et al., 1987) G12 item (‘lack of judgement in insight’) was collected as part of the routine clinical assessment and is therefore included to provide a rating of insight collected by a researcher blind to other data. The PANSS G12 item is scored on a scale of 1 (full insight) to 7 (maximal lack of insight).

#### 2.2.2 Distress

Distress was indexed as depression, anxiety, suicidal ideation, and (low) self-esteem. Depression was measured using the Beck Depression Inventory — 2nd edition (BDI-II, Beck et al., 1996), a widely used 21-item self-report questionnaire which includes minor revisions of the original BDI (Beck et al., 1961) to bring it into line with the current diagnostic criteria. Anxiety was measured using the Beck Anxiety Inventory (BAI, Beck et al., 1988). The Beck Scale for Suicide Ideation (BSS, Beck et al., 1979) was used to measure severity of suicidal ideation. Self esteem was assessed using the Rosenberg Self-Esteem Scale (RSE, Rosenberg, 1965), a self-report measure of feelings of self-worth or self-acceptance. Higher RSE scores indicate poorer self-esteem.

#### 2.2.3 Coping styles

The COPE (Carver et al., 1989) is a multi-dimensional coping inventory which assesses the ways in which individuals respond to stress. For this study, each item (e.g. ‘I've been trying to get emotional support from friends or relatives’) was answered in terms of how much the participant had been doing what was described since they had been told that they had a mental health problem, ignoring whether it was successful or not, on a four-point scale (not at all, a little, a medium amount, a lot). The COPE scale is made up of 60 items, from which the following 15 subscales (four items each; possible score range 0 to 12) are derived:

**Table:** 

1.	Positive reinterpretation
2.	Mental disengagement
3.	Venting of emotions
4.	Use of instrumental social support
5.	Active coping
6.	Denial
7.	Religious coping
8.	Humour
9.	Behavioural disengagement
10.	Restraint
11.	Use of emotional social support
12.	Substance use
13.	Acceptance
14.	Suppression of competing activities
15.	Planning

The raw scores for each coping subscale are likely to be influenced by each individual's response bias for the questionnaire (i.e. overall tendency to agree or disagree with the statements). Following Lysaker et al. (2003b) approach, relative preference for each COPE subscale (i.e. the tendency to endorse items for that subscale relative to overall endorsement level) was calculated by dividing the score for each COPE subscale by the total COPE score. Each relative preference score was then multiplied by 15 to give a set of relative preference scores for each individual which had an overall mean of 1.00.

### 2.3 Statistical analyses

#### 2.3.1 Dimensions of insight: factor analysis of insight measures

The way in which the items of the SAI-E should be combined into dimensions of insight is not clear (as mentioned earlier). A factor analysis of the insight items was therefore conducted in order to determine how the BIS and SAI-E could best be combined into meaningful dimensional scores.

#### 2.3.2 Correlations between insight, distress and coping styles

Parametric statistics were applied to variables which approximated normal distributions. This was judged by the inspection of histograms and examination of skewness statistics. Where variables were not distributed normally, non-parametric correlations (Spearman's rho) were used.

Relationships between total insight score, insight dimensions, and measures of distress were not corrected for multiple comparisons because they were hypothesised a priori and have been found in a substantial number of previous studies (Carroll et al., 1999, Carroll et al., 2004, Cunningham Owens et al., 2001, Schwartz, 2001, Mintz et al., 2003, Schwartz and Smith, 2004, Drake et al., 2004). Furthermore, the relationships between total insight score, insight dimensions, and coping styles that were hypothesised a priori were not corrected for multiple comparisons. Correlations between insight dimensions and particular styles of coping which had not been hypothesised a-priori were re-considered after correction using the false discovery rate (FDR, Benjamini and Hochberg, 1995) method. This allows the average proportion of false rejections of the null hypothesis (type I errors) to be controlled without being as overly conservative as the Bonferroni method, which would increase the likelihood of making type II errors by incorrectly not rejecting the null hypothesis (false-negative). Finally, the relationships of particular coping styles that correlated with insight to measures of distress were explored to gain further understanding into the insight-coping styles relationships.

## 3 Results

### 3.1 Factor analysis of insight measures

Data were available on all items of the BIS and SAI-E for 57 participants. Results of the factor analysis indicated that the Kaiser–Meyer–Olkin measure of sampling adequacy was at an acceptable level (0.683). Bartlett's test of sphericity was highly significant (*χ*^2^ = 379.8, *df* = 105, *p* < 0.001), indicating that all correlations tested simultaneously were significantly different from zero.

In the principal components analysis, four factors with an eigenvalue greater than one accounted for 64.3% of the total variance were extracted and rotated to an orthogonal criterion. The Varimax solution converged in six iterations. Table 2 shows these rotated factor loadings.

**Table 2:** Loadings (> 0.50 shown in bold) for the rotated factor analysis solution for insight items

Insight item		Factor			
		1	2	3	4
SAI-E q4	“How do you explain your condition/disorder/illness”	**0.844**	0.117	0.002	0.181
SAI-E q3	“Do you think your condition amounts to a mental illness or mental disorder?”	**0.823**	0.284	− 0.027	− 0.058
SAI-E q5	“Has your condition (use patient's term) led to adverse consequences or problems in your life?”	**0.735**	− 0.048	0.292	0.017
BIS q8	“None of the unusual things I experience are due to an illness”	**0.604**	0.328	0.205	0.230
SAI-E q8	Attribution of symptoms	**0.577**	0.325	0.301	0.053
BIS q2	“I am mentally well”	**0.570**	− 0.009	0.146	0.257
BIS q7	“If somebody said that I have a nervous or a mental illness then they would be right”	**0.551**	0.236	0.352	0.011
BIS q5	“The doctor is right in prescribing medication for me”	− 0.007	**0.824**	− 0.006	0.002
BIS q3	“I do not need medication”	0.264	**0.769**	0.064	0.190
SAI-E q6	““Do you think your condition (use patient's term) or the problem resulting from it needs treatment?”	0.401	**0.627**	0.202	− 0.115
SAI-E q2	“Do you think this means there is something wrong with you?”	0.278	− 0.187	**0.825**	0.075
SAI-E q1	“Do you think you have been experiencing any emotional or psychological changes or difficulties?”	0.099	0.177	**0.807**	0.001
BIS q6	“I do not need to be seen by a doctor or psychiatrist”	0.164	0.451	**0.566**	0.086
BIS q1	“Some of my symptoms are made by my mind”	0.137	0.005	− 0.069	**0.894**
SAI-E q7	Awareness of symptoms	0.151	0.150	0.473	**0.613**

SAI-E = Schedule for the Assessment of Insight — Expanded. BIS = Birchwood Insight Scale.

Insight factor scores were developed based on the factor loadings and were used in all subsequent analyses of insight data. For each item, the highest factor loading determined inclusion in a factor score.

Factor 1 included items relating to awareness of illness, together with the attribution of problems in general as well as symptoms specifically to a mental illness. It is possible for an individual to be aware that his/her symptomatic experiences are abnormal/generated by his/her mind but not attribute them to a mental illness and therefore score highly on factor 4 (see further) but not on factor 1. The score derived from these factor loadings was therefore named ‘Awareness of and Attribution to Illness’ (AAI), and has a potential range of 0 to 16. Factor 2 included items relating to awareness of the need for treatment, specifically medication. The score derived from these factor loadings was therefore named ‘Recognition of the Need for Medication’ (RNM), and has a potential range of 0 to 6. The items loading on factor 3 related to awareness of problems and the need to seek help. The score derived from these factor loadings was therefore named ‘Awareness of Problems’ (AP), and has a potential range of 0 to 6. To clarify further the nature of factors 2 (RMN) and 3 (AP), the items which loaded on factor 2 referred to being aware that one needs to take medication, whereas the items which loaded on factor 3 referred to the awareness of the need to seek help more generally, and being aware that there was ‘something wrong’. Finally, factor 4 comprised items relating to awareness of symptoms. These items referred to the ability to recognise that experiences are abnormal and label them as such but not perceive them as a problem or an illness. The score derived from these factor loadings was therefore named ‘Symptom Re-labelling’ (SR), and has a potential range of 0 to 6.

### 3.2 Descriptive statistics

#### 3.2.1 Insight factors derived from factor analysis

The insight items for all participants were combined according to the loadings derived from the factor analysis. All 65 participants had the data necessary for calculating the awareness of problems (AP) factor score. One participant declined to answer one of the questions relating to the need for medication, and so a score for recognition of the need for medication (RNM) could not be derived, leaving 64 participants with this score. Eight participants could not be rated for either the awareness or attribution of symptoms on the SAI-E (because they were asymptomatic), preventing scores from being derived for the awareness of and attribution to illness (AAI) and symptom re-labelling (SR) factors for these patients. Table 3 presents the descriptive statistics for the total insight scores on the SAE-I and the BIS, and the insight factors derived from the factor analysis.

**Table 3:** Descriptive statistics for measures of insight, distress and coping

	*N* (missing)	Mean	SD	Range	Skewness
*Insight (total)*					
SAE-E	55 (10)	12.38	5.51	0–20	− 0.53
BIS	64 (1)	8.63	2.96	1–12	− 0.70

*Insight factors*					
AAI	57 (8)	9.86	4.95	0–16	− 0.56
RNM	64 (1)	4.78	1.69	0–6	− 1.41
AP	65 (0)	4.38	1.95	0–6	− 0.86
SR	57 (8)	3.37	1.95	0–6	− 0.31

*Distress*					
Beck Depression Inventory — II	64 (1)	15.12	11.87	0–54	0.72
Beck Anxiety Inventory	65 (0)	14.46	11.70	0–52	1.07
Beck Scale for Suicide Ideation	65 (0)	2.94	6.73	0–37	**3.13**
Rosenberg Self-Esteem Scale	64 (1)	22.78	6.73	10–36	− 0.05

*Coping*^⁎^					
Positive reinterpretation	55 (10)	1.31	0.53	0–2.39	− 0.48
Mental disengagement	55 (10)	1.24	0.64	0–3.33	0.69
Venting of emotions	55 (10)	0.93	0.73	0–3.75	1.41
Use of instrumental social support	55 (10)	1.14	0.45	0–2.39	− 0.16
Active coping	55 (10)	1.15	0.57	0–2.18	− 0.65
Denial	55 (10)	0.64	0.61	0–3.06	1.69
Religious coping	55 (10)	1.26	0.95	0–4.86	0.94
Humour	55 (10)	0.70	0.66	0–3.21	1.12
Behavioural disengagement	55 (10)	0.56	0.50	0–1.86	0.43
Restraint	55 (10)	0.86	0.54	0–2.14	0.16
Use of emotional social support	55 (10)	1.19	0.64	0–2.78	0.33
Substance use	55 (10)	0.61	0.99	0–4.19	**2.25**
Acceptance	55 (10)	1.51	0.63	0–2.95	− 0.06
Suppression of competing activities	55 (10)	0.89	0.50	0–1.89	− 0.33
Planning	55 (10)	1.02	0.55	0–2.02	− 0.26

AAI — awareness of and attribution to illness. RNM — recognition of the need for medication. AP — awareness of problems. SR — symptom re-labelling.

The AAI and SR factor scores met the criteria for the use of parametric statistics. Inspection of the histograms for the RNM and AP factors indicated that the data for both factors were not normally distributed. Non-parametric Spearman's rho correlations were therefore used to examine the correlations of these factors.

In order to assess the convergent validity of the insight factors, correlations between these scores and the PANSS G12 insight item were examined. As expected, all four sub-scales were significantly negatively correlated with the PANSS G12 item (which is reverse scored). The AAI factor score showed the strongest correlation (*r* = − 0.547, *p* < 0.001), closely followed by the RNM factor score (rho = − 0.503, *p* < 0.001). The AP and SR factor scores showed significant, but slightly weaker associations (rho = − 0.307, *p* < 0.05) and (r = − 0.293, *p* < 0.05) respectively.

#### 3.2.2 Distress measures

The descriptive statistics for the four measures of distress included in this study are presented in Table 3. The BSS was highly negatively skewed and inspection of the histogram indicated that the data were not normally distributed. Non-parametric statistics were therefore applied to this measure, while parametric statistics were applied to the remaining three distress measures.

The mean score of 15.12 on the BDI-II indicated mild depression, while the mean score of 14.46 on the BAI indicated mild-to-moderate anxiety. The mean score of 22.78 on the Rosenberg self-esteem scale indicated a moderate level of self-esteem.

#### 3.2.3 Coping styles

The descriptive statistics for relative preference for each of the COPE subscales are shown in Table 3. The ‘substance use’ subscale was positively skewed and inspection of its histogram indicated that the data were not normally distributed. Non-parametric statistics were therefore applied to this subscale, while parametric statistics were applied to the remaining 14 subscales.

### 3.3 Correlations between insight and distress

There was a trend for an association between lower insight, as assessed with the total score on the BIS, and poor self-esteem (*r* = 0.227, 0 = 0.07). At the factor level, the SR factor was positively correlated with BDI-II depression (*r* = 0.273, *p* < 0.05) and a positive association with poor self-esteem approached significance (*r* = 0.257, *p* = 0.054). The AP factor was positively correlated with both BDI-II depression (rho = 0.308, *p* < 0.05) and BAI anxiety (rho = 0.293, *p* < 0.05).

### 3.4 Correlations between insight and coping styles

Table 4 shows the correlations between insight and preference for the COPE subscales.

**Table 4:** Correlations between insight and coping style

COPE subscale	SAE-I (total)	BIS (total)	Insight factor			
			AAI (*n* = 47)	RNM (*n* = 54)	AP (*n* = 55)	SR (*n* = 47)
Positive reinterpretation and growth	− 0.288⁎	− 0.195	− 0.186	− *0.031*	− *0.195*	**− **0.323⁎
Mental disengagement	− 0.275	− 0.109	− 0.124	− *0.091*	− *0.338*⁎	− 0.084
Focus on venting emotions	− 0.049	− 0.220	− 0.087	*0.040*	− *0.044*	− 0.214
Use of instrumental social support	0.365⁎	0.195	0.458⁎⁎	*0.075*	*0.149*	− 0.056
Active coping	0.175	0.003	0.176	*0.054*	*0.067*	− 0.058
Denial	0.113	0.051	0.067	*0.040*	− *0.029*	0.249
Religious coping	0.100	0.161	0.155	*0.147*	− *0.034*	− 0.045
Humour	− 0.399^a^	− 0.216	**− **0.311^a^	− *0.135*	− *0.271*	− 0.230
Behavioural disengagement	− 0.194	− 0.16	**− **0.301⁎	− *0.067*	*0.222*	0.098
Restraint	− 0.023	− 0.144	− 0.222	− *0.129*	*0.155*	0.217
Use of emotional social support	0.120	0.335⁎	0.219	*0.238*	*0.166*	0.034
Substance use	− 0.146	− *0.105*	− *0.146*	− *0.127*	− *0.190*	*0.018*
Acceptance	− 0.126	0.075	− 0.099	− *0.049*	*0.123*	− 0.028
Suppressing competing activities	0.214	0.135	0.129	*0.094*	*0.281*⁎	0.305⁎
Planning	0.413⁎	0.085	0.317⁎	− *0.059*	*0.267*	0.198

Non-parametric correlations in italics. AAI — awareness of and attribution to illness. RNM — recognition of the need for medication. AP — awareness of problems. SR — symptom re-labelling.

a Not significant after excluding one outlier subject.

⁎ *p* < 0.05.

⁎⁎ *p* < 0.001.

Total insight score on the SAI-E was correlated with three COPE subscales: ‘positive reinterpretation and growth’ (*r* = − 0.288, *p* < 0.05), ‘use of instrumental social support’ (*r* = 0.365, *p* < 0.05) and ‘planning’ (*r* = − 0.413, *p* < 0.05). The direction of association of these copying subscales with the total insight score on the BIS was in the same direction as noted on the SAE-I but somewhat weaker in strength. In addition, the total insight score on the BIS correlated positively with ‘use of social emotional support’ (*r* = 0.335, *p* < 0.05).

At the factor level, scores on the AAI insight factor were positively correlated with preference for two COPE subscales: ‘use of instrumental social support’ (*r* = 0.458, *p* < 0.001) and ‘planning’ (*r* = 0.317, *p* < 0.05). As hypothesised, AAI score was negatively correlated with preference for ‘behavioural disengagement’ (*r* = − 0.301, *p* < 0.05). It was also negatively correlated with ‘humour’ (*r* = − 0.311, *p* < 0.05), but this relationship was not significant when a high score extreme value identified in the scatter plot was excluded (*r* = − 0.220, ns). AP insight factor scores were negatively correlated with preference for ‘mental disengagement’ (rho = − 0.346, *p* < 0.05). Scores on the SR insight factor were negatively correlated with preference for ‘positive reinterpretation and growth’ (*r* = − 0.323, *p* < 0.05). They were also positively correlated with ‘suppression of competing activities’ (*r* = 0.305, *p* < 0.05). Of the correlations that were not predicted in advance, the correlation between the AAI component of insight and preference for the use of instrumental social support survived FDR correction and explained 21% of the variance.

### 3.5 Correlations between coping styles and distress

Supporting our predictions, the preference for ‘positive reinterpretation and growth’ as a coping style was negatively correlated with depression (*r* = − 0.439, *n* = 55, *p* < 0.001) and poor self-esteem (*r* = − 0.350, *p* < 0.01) while ‘behavioural disengagement’ as a coping style was positively correlated with depression (*r* = 0.457, *p* < 0.001), anxiety (*r* = 0.382, *p* < 0.005), and poor self-esteem (*r* = 0.386, *p* < 0.005).

## 4 Discussion

Previous literature suggests that better insight is directly related to greater distress (Carroll et al., 1999, Carroll et al., 2004, Cunningham Owens et al., 2001, Schwartz, 2001, Mintz et al., 2003, Schwartz and Smith, 2004, Drake et al., 2004) and that poor insight in schizophrenia may be associated with the use of denial as a coping style (Moore et al., 1999). However, the relationships between insight and coping styles beyond denial have seldom been investigated. This investigation aimed to extend the literature on the psychological denial model of insight by examining the relationships between insight, measures of distress, and a comprehensive measure of coping style.

We found that poor insight (total score on the BIS) was associated with less distress but only at a trend level. The focus of our study, however, was on the multi-factorial nature of insight. The results at the factor level revealed that the association between insight and distress was true only for two of the four insight factors. Specifically, symptom re-labelling (SR) and awareness of problems (AP) were related to depression, and AP was also related to anxiety. There was a further strong trend (*p* = .054) between SR and self-esteem. However, no support was found for the hypothesised relationship between poor insight and ‘preference for denial’ as a coping style, or between distress and denial. This hypothesis was based on the findings of previous studies which used different coping style scales, which often only measured denial (Moore et al., 1999, Young et al., 1998, Lysaker et al., 2005a), rather than the wide range of coping styles measured by the COPE. Specifically, Moore et al. (1999) used the Balanced Inventory of Desirable Responding. This measure deals specifically with two aspects of denial, ‘self-deceptive positivity’ and ‘impression management’. As such, it provides a detailed measure of denial, but does not index the wide range of coping styles measured by the COPE. Similarly, the ‘denial items’ from the Minnesota Multiphasic Personality Inventory were used as the measure of denial by Young et al. (1998). However, these were not designed specifically to measure the use of denial as a coping style, but rather assess a person's ability to acknowledge ‘minor personal failings of virtually universal proportion’ such as ‘I gossip a little at times’. The only study to use a measure of multiple coping styles which reported a relationship between insight and a coping style aligned with denial (distancing from the WCQ) (Lysaker et al., 2003b) showed that denial plays a role in the unawareness of illness but only in a sub-group of patients with schizophrenia who possess good executive function. It is also possible that the COPE does not measure the concept of ‘denial’ in sufficient depth for relationships with insight to be detected, or that differences in the way the concept is operationalised in different scales may lead to the discrepant findings. However, other styles of coping related meaningfully to insight and distress in this study.

As expected, a negative relationship was found between preference for ‘positive reinterpretation and growth’ as a coping style and the SR insight factor. This replicates finding of Lysaker et al. (2003a) of an association between preference for ‘positive reinterpretation’ (from the WCQ) and lower awareness of symptoms (from the Schedule to assess Unawareness of Mental Disorders, Amador et al., 1993). It therefore appears possible to detect this specific relationship using different measures of insight and different measures of coping style, suggesting it is more robust than the relationship between insight and denial. Furthermore, a preference for ‘positive reinterpretation and growth’ was negatively correlated with both depression and poor self-esteem. These findings suggest that adopting a positive reinterpretation style of coping, rather than a denial style of coping, is the significant factor involved in reducing distress. Positively reframing unusual mental experiences may enable individuals to find meaning in their symptoms outside of the medical model, for example by attributing a spiritual meaning to their experiences. Indeed, Brett (2004) found that spiritual appraisals of anomalous experiences in two groups of individuals with and without a need for care were predictive of less distress, while medical model appraisals were predictive of more distress. Taken together, these findings further support the notion that, in some circumstances at least, ‘poor insight’ (in terms of rejecting a medical model explanation), may be adaptive (McGorry and McConville, 1999, Lysaker et al., 2005b). In a similar vein, Lysaker et al. (2005b) suggest that constructing a personal understanding in which symptoms are not assigned great importance, and where personal strengths are acknowledged, may help individuals to ward off the ‘misery and social isolation’ of schizophrenia. More recently, Lysaker et al. (2007) have shown that patients with high insight but minimum acceptance of stigmatizing beliefs have less impaired social function than those with high/low insight but mild-to-moderate acceptance of stigmatizing beliefs. These findings are in line with the recent recovery model, which emphasises that the important factor towards recovery is not cure or acceptance of illness, but rather people coming to their own understanding of their illness and reintegration into their communities (May, 2004).

A negative relationship between ‘behavioural disengagement’ and illness awareness (AAI) insight factor also emerged. The items which make up the ‘behavioural disengagement’ subscale deal with ‘giving up’, such as ‘I admit to myself that I can't deal with it, and quit trying’ (Carver et al., 1989). As can be expected, this coping style was also associated with greater distress, as measured by depression, anxiety, and poor self-esteem. It is possible that adopting an avoidant coping style causes distress and decreases insight in terms of lower (reported) awareness of the illness. Regardless of the direction of causality in these relationships, low awareness of illness appears ‘maladaptive’ when accompanied by an avoidant coping style. Behavioural disengagement could perhaps be targeted in cognitive behavioural interventions, with the dual aims of decreasing distress and improving insight.

A robust relationship was found between the AAI insight factor and preference for the ‘use of instrumental social support’ as a coping style. The items for this COPE sub-scale address discussing ‘the problem’ (in this case a mental health problem) with other people in order to gather information and decide what to do about it. This relationship suggests that people who are aware that they have a mental illness are also less isolated. This result concords with previous findings linking poor insight to poor interpersonal functioning (Lysaker et al., 1998). The direction of causality in this relationship is unclear. One possibility may be that people have better insight because they discuss their mental health problems with others, or, alternatively, that people who accept that they have a mental health problem are more likely to seek support for it. In either case, getting external input about a mental health problem appears to be associated with better insight, making it a potential avenue for intervention (Dolder et al., 2003). Importantly, the ‘use of instrumental social support’ related to better insight but not to (high) distress. This finding is in line with the stress-buffering role of social support, acting as a protective moderator between the experience of stressful life events and depression (Windle, 1992).

With replication, the observations of this study may have theoretical and practical implications. At the theoretical level, our observations offer support to the models of recovery from schizophrenia which emphasize the importance of adaptive narratives of self and illness re-appraisal as the key for many affected individuals in moving towards wellness (Levy et al., 1975, McGlashan and Carpenter, 1981, Warner, 1994, McGorry and McConville, 1999, May, 2004). At the practical level, they advocate the use of tailored behavioural interventions aimed specifically at facilitation of active coping, for example seeking opportunities to talk and learn about the psychotic experiences, especially in those with high insight on the AAI sub-factor and high distress and, in parallel, development of positive narratives of the self and illness combating self-stigmatizing beliefs to aid recovery at least in the domains of social functioning and hopefulness for the future (Resnick et al., 2004).

### 4.1 Limitations

The study has a number of limitations. First, this was a cross-sectional study and fails to inform about the direction of causality in the observed insight-distress-coping style relationships. We were not able to discount alternatives explanations for our findings, specifically that depression may produce an increased tendency to accept symptoms and problems, rather than the awareness of symptoms and problems leading to depression and low self-esteem; lower depression may predispose people to the coping style of positive reinterpretation and growth rather than this particular coping style leading to reduced distress; and a lack of inclination, rather than lack of opportunity, may lead to the failure to use instrumental social support. Second, we had performed a large number of correlations on data from a sample of 57 patients. Some of our observations may represent chance findings. On the other hand, the relationships that were present but failed to survive correction for multiple comparisons were not discussed. It is possible that they represent true effects of small size and not necessarily spurious findings. Nevertheless, we have reported the strength of all such correlations so that they could be considered further in future studies. Third, almost all the relationships are weak and may be mediated by other variables, for example executive functioning (for review see Aleman et al., 2006, Cooke et al., 2005, Lysaker et al., 2006), which have known associations with insight but were not examined in this investigation. Finally, the failure to find support for the hypothesised relationships between poor insight and the use of denial as a coping style may be due to lack of consideration of executive/general cognitive functioning as a potential mediator of this relationship (Lysaker et al., 2003b) or to the measure of denial that was used.

## 5 Conclusions

The findings of this study support the position that possessing good insight, specifically in terms of being aware of having a mental illness and associated problems, is associated with greater distress in schizophrenia. They do not, however, support the psychological denial explanation for this relationship. Rather, a complex pattern of relationships between different dimensions of insight, coping styles, and distress emerged. Preference for positive reinterpretation was related to both lower distress and lower symptom awareness (SR factor), while ‘preference for mental disengagement’, an avoidant coping style, was associated with lower insight (AAI factor) but greater distress. Finally, a strong relationship between better insight (AAI factor) and ‘preference for a social support-seeking’ coping style emerged, although the latter was not related to distress. These findings call into question the assumption that having poor insight is always maladaptive, and suggest that a positive reinterpretation style and more active coping such as discussing a mental health problem with others may be adaptive responses to a diagnosis of schizophrenia. Furthermore, the findings advocate the use of a dimensional approach to examine the potential value of insight as a therapeutic target with a view to aid recovery and improve functional outcome in individuals affected with schizophrenia.

## 6 Role of the Funding Source

The sponsor (the Wellcome Trust) had no role in study design; in the collection, analysis and interpretation of data; in the writing of the report; or in the decision to submit the paper for publication.

## 7 Contributors

Veena Kumari, Elizabeth Kuipers, Emmanuelle Peters and Michael Cooke designed the study and obtained the funding. Michael Cooke and Ingrid Aasen collected the data. Dominic Fannon and Anantha Anilkumar performed the clinical diagnostic interviews. Michael Cooke undertook the statistical analysis and prepared the first draft. All authors contributed to and approved the final manuscript.

## 8 Conflict of Interest

The authors declare no conflict of interest.
